# Decline in Applicants to Obstetrics and Gynecology Fellowships: Influence on Match Rates and Competitiveness

**DOI:** 10.1097/og9.0000000000000153

**Published:** 2026-02-19

**Authors:** Leigh A. Humphries, Divya K. Shah, Anthony L. Shanks, Lisa G. Hofler, Lorene A. Temming, Stephanie N. Morris, Sarah K. Dotters-Katz

**Affiliations:** Center for Minimally Invasive Gynecologic Surgery, Newton-Wellesley Hospital & Mass General Brigham, Harvard Medical School, Newton, Massachusetts; Department of Obstetrics and Gynecology, Division of Reproductive Endocrinology and Infertility, Hospital of the University of Pennsylvania, Philadelphia, Pennsylvania; Department of Obstetrics and Gynecology, Division of Maternal Fetal Medicine, Indiana University School of Medicine, Indianapolis, Indiana; Department of Obstetrics and Gynecology, Division of Complex Family Planning, University of New Mexico, Albuquerque, New Mexico; Department of Obstetrics and Gynecology, Division of Maternal-Fetal Medicine, Wake Forest University School of Medicine, Atrium Health Carolinas Medical Center, Charlotte, North Carolina; and Department of Obstetrics and Gynecology, Division of Maternal-Fetal Medicine, Duke University, Durham, North Carolina.

## Abstract

Although applications to fellowships in obstetrics and gynecology have declined in recent years, match rates have increased as most programs continue to fill available positions.

Fellowship training is the primary pathway through which obstetrician–gynecologists enter the subspecialty workforce. Maintaining this pipeline is essential amid the increasing technical complexity of the field and the demand for specialized expertise. Seven obstetrics and gynecology subspecialties currently participate in the National Resident Matching Program Match: gynecologic oncology, maternal–fetal medicine, reproductive endocrinology and infertility, urogynecology and reconstructive pelvic surgery, minimally invasive gynecologic surgery, pediatric and adolescent gynecology, and complex family planning.^1^ Each year, hundreds of residents apply to these fellowships, requiring substantial time and resources from applicants, programs, and sponsoring institutions. Longitudinal analysis of National Resident Matching Program data is needed to evaluate trends in match outcomes across subspecialties. Recently, some fellowship administrators have anecdotally reported fewer applications to their programs. Changes in the applicant pool could affect the efficiency of the recruitment process and future availability of subspecialists. This study reviews 12 years of National Resident Matching Program data 1) to quantify fellowship applicants relative to graduating residents per year, 2) to describe changes in match and program fill rates by subspecialty, and 3) to compare subspecialty competitiveness.

## METHODS

This study was deemed exempt by the IRB because it did not meet the criteria for human subject research. This cross-sectional study analyzes annual National Resident Matching Program data on obstetrics and gynecology fellowship applicants and programs from 2014 to 2025.^1^ Complex family planning data were available starting in 2021, the first year of complex family planning participation in the Match. Data on graduating obstetrics and gynecology residents were extracted from the Accreditation Council for Graduate Medical Education Data Resource Book.^2^ Outcomes included the number of applicants, programs, and positions per year by subspecialty; match and program fill rates; proportion of graduating residents who applied and matched to fellowship; and subspecialty competitiveness. Match rate was the proportion of applicants who successfully matched, and program fill rate was the proportion of programs that filled all positions. Competitiveness was measured by the Normalized Competitive Index, calculated as program fill rate divided by match rate, normalized across subspecialties each year.^3,4^ Trends were analyzed with Cochrane–Armitage tests for proportions and linear regression for continuous variables. Regression assumptions (linearity, independence, homoscedasticity) were assessed and met.

## RESULTS

**Fig. 1.**
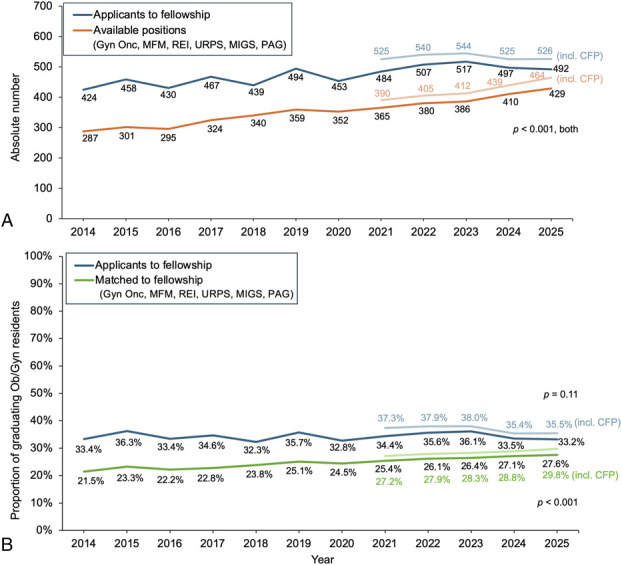
Trends in seven obstetrics and gynecology subspecialty fellowships, 2014 to 2025. Number of applicants and available positions per year **(A)** and estimated proportion of graduating U.S. obstetrics and gynecology residents applying to and matching to fellowship **(B)**. Gyn Onc, gynecologic oncology; MFM, maternal–fetal medicine; REI, reproductive endocrinology and infertility; URPS, urogynecology and reconstructive pelvic surgery; MIGS, minimally invasive gynecologic surgery; PAG, pediatric and adolescent gynecology; CFP, complex family planning.

The number of applicants to obstetrics and gynecology fellowships increased overall from 2014 to 2023, paralleling steady growth in total positions (*P*<.001) (Fig. 1A). However, from 2023 to 2025, the number of applicants declined slightly and plateaued, despite a continued rise in positions. As a result, the proportion of obstetrics and gynecology residents applying to fellowship, which peaked at 38.0% in 2023, declined to 35.5% in 2025 (Fig. 1B). Correspondingly, the applicant-to-position ratio decreased from 1.7 in 2014 to 1.1 in 2025, reflecting the narrowing gap between the number of available positions and applicants across subspecialties (Appendix 1, available online at http://links.lww.com/AOG/E537). By 2025, these ratios for gynecologic oncology, maternal–fetal medicine, and complex family planning reached 1.0; ie, there were just enough applicants to fill the positions. Applicant-to-position ratios for urogynecology and reconstructive pelvic surgery, pediatric and adolescent gynecology, minimally invasive gynecologic surgery, and reproductive endocrinology and infertility fluctuated from 1.2 to 1.6.

*Humphries. Decline in Applicants to Obstetrics and Gynecology Fellowships. O&G Open 2026*.

With fewer applicants to compete for positions, the overall fellowship match rate increased significantly from 21.5% of obstetrics and gynecology residents in 2014 to 29.8% in 2025 (*P*<.001, Fig. 1B). By 2025, subspecialty-specific match rates exceeded 90% in gynecologic oncology, maternal–fetal medicine, and complex family planning, with significant linear trends over time for gynecologic oncology, maternal–fetal medicine, minimally invasive gynecologic surgery, and complex family planning (*P*<.01, Appendix 2, http://links.lww.com/AOG/E537). Programs continued to fill nearly all positions (typically 95–100%) (Appendix 3, http://links.lww.com/AOG/E537).

**Fig. 2.**
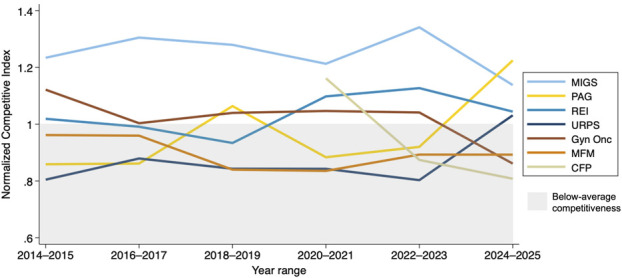
Normalized Competitive Index score across obstetrics and gynecology subspecialties, averaged over 2-year intervals. Values greater than 1.0 indicate above-average competitiveness, and values less than 1.0 indicate below-average competitiveness. MIGS, minimally invasive gynecologic surgery; PAG, pediatric and adolescent gynecology; REI, reproductive endocrinology and infertility; URPS, urogynecology and reconstructive pelvic surgery; Gyn Onc, gynecologic oncology; MFM, maternal–fetal medicine; CFP, complex family planning.

Because program fill rates remained high, subspecialty competitiveness was driven largely by differences in match rates (Fig. 2). As match rates rose from 2023 to 2025 in gynecologic oncology, maternal–fetal medicine, minimally invasive gynecologic surgery, and reproductive endocrinology and infertility, their relative competitiveness dropped. In contrast, urogynecology and reconstructive pelvic surgery and pediatric and adolescent gynecology experienced modest increases in competitiveness recently; their match rates did not increase significantly or declined. Minimally invasive gynecologic surgery was the most competitive of the seven subspecialties overall, with a Normalized Competitive Index score consistently above 1.0. In recent years, complex family planning has become the least competitive fellowship.

*Humphries. Decline in Applicants to Obstetrics and Gynecology Fellowships. O&G Open 2026*.

## DISCUSSION

Because most fellowship applicants apply directly from residency, we estimate that about one-third of obstetrics and gynecology residents apply to fellowship each year, with most successfully matching. However, after nearly a decade of growth, the number of fellowship applicants has plateaued or declined in recent years across most obstetrics and gynecology subspecialties. Fellowship programs have continued to expand and fill their positions, despite a smaller applicant pool; thus, match rates have increased significantly, particularly in gynecologic oncology, maternal–fetal medicine, minimally invasive gynecologic surgery, and complex family planning. These changes in match rates have directly influenced the “competitiveness” of each subspecialty, ie, its ability to effectively recruit applicants and fill positions.

As the applicant-to-position ratio nears 1.0 across several obstetrics and gynecology subspecialties, programs may face challenges filling positions if fellowship growth continues to outpace applicant demand. The reasons for the plateauing number of applicants are unclear but may reflect shifting priorities related to lifestyle, compensation, job availability, and burnout.^5–7^ Although this study does not capture individual-level factors influencing Match participation or success, it demonstrates from aggregate national data that the overall fellowship pipeline remains stable. Continued monitoring is needed to ensure that program expansion aligns with applicant interest and clinical needs.
